# Biochemical and virological analysis of the 18-residue C-terminal tail of HIV-1 integrase

**DOI:** 10.1186/1742-4690-6-94

**Published:** 2009-10-19

**Authors:** Mohd J Dar, Blandine Monel, Lavanya Krishnan, Ming-Chieh Shun, Francesca Di Nunzio, Dag E Helland, Alan Engelman

**Affiliations:** 1 Department of Cancer Immunology and AIDS, Dana-Farber Cancer Institute, 44 Binney Street, Boston, MA, USA; 2 Molecular Biology Institute, University of Bergen, N-5020 Bergen, Norway; 3 Current Address: University of Pittsburgh School of Medicine, S-427 BST, 200 Lothrop Street, Pittsburgh, PA 15213, USA

## Abstract

**Background:**

The 18 residue tail abutting the SH3 fold that comprises the heart of the C-terminal domain is the only part of HIV-1 integrase yet to be visualized by structural biology. To ascertain the role of the tail region in integrase function and HIV-1 replication, a set of deletion mutants that successively lacked three amino acids was constructed and analyzed in a variety of biochemical and virus infection assays. HIV-1/2 chimers, which harbored the analogous 23-mer HIV-2 tail in place of the HIV-1 sequence, were also studied. Because integrase mutations can affect steps in the replication cycle other than integration, defective mutant viruses were tested for integrase protein content and reverse transcription in addition to integration. The F185K core domain mutation, which increases integrase protein solubility, was furthermore analyzed in a subset of mutants.

**Results:**

Purified proteins were assessed for in vitro levels of 3' processing and DNA strand transfer activities whereas HIV-1 infectivity was measured using luciferase reporter viruses. Deletions lacking up to 9 amino acids (1-285, 1-282, and 1-279) displayed near wild-type activities *in vitro *and during infection. Further deletion yielded two viruses, HIV-1_1-276 _and HIV-1_1-273_, that displayed approximately two and 5-fold infectivity defects, respectively, due to reduced integrase function. Deletion mutant HIV-1_1-270 _and the HIV-1/2 chimera were non-infectious and displayed approximately 3 to 4-fold reverse transcription in addition to severe integration defects. Removal of four additional residues, which encompassed the C-terminal β strand of the SH3 fold, further compromised integrase incorporation into virions and reverse transcription.

**Conclusion:**

HIV-1_1-270_, HIV-1_1-266_, and the HIV-1/2 chimera were typed as class II mutant viruses due to their pleiotropic replication defects. We speculate that residues 271-273 might play a role in mediating the known integrase-reverse transcriptase interaction, as their removal unveiled a reverse transcription defect. The F185K mutation reduced the *in vitro *activities of 1-279 and 1-276 integrases by about 25%. Mutant proteins 1-279/F185K and 1-276/F185K are therefore highlighted as potential structural biology candidates, whereas further deleted tail variants (1-273/F185K or 1-270/F185K) are less desirable due to marginal or undetectable levels of integrase function.

## Background

Retrovirus replication proceeds through a series of steps that initiate upon virus entry into a cell, followed by particle uncoating and reverse transcription. To support productive replication, the resulting double stranded cDNA must be integrated into a cell chromosome. The integrated DNA provides an efficient transcriptional template for viral gene expression and ensures for segregation of viral genetic material to daughter cells during division. Due to its essential nature, the integrase (IN) encoded by HIV-1 is an intensely studied antiviral drug target [1].

Integration can be divided into three enzyme-based steps, the first two of which are catalyzed by IN. In the initial 3' processing reaction, IN removes the terminal pGT_OH _dinucleotides from the 3' ends of the blunt-ended HIV-1 reverse transcript, yielding the precursor ends for integration [2-4]. In the second step, DNA strand transfer, IN uses the 3'-oxygens to cut the chromosomal target DNA in a staggered fashion and at the same time joins the viral 3' ends to the resulting 5' phosphates [3]. The final step, repair of single stranded gaps and joining of viral DNA 5' ends, is accomplished by cellular enzymes [5,6]. HIV-1 IN activities can be measured in vitro using oligonucleotide DNA substrates that mimic the ends of the reverse transcript and either Mg^2+ ^or Mn^2+ ^cofactor [7-10].

IN is a multi-domain protein consisting of the N-terminal domain (NTD, HIV-1 residues 1-49), catalytic core domain (CCD, residues 50-212), and C-terminal domain (CTD, residues 213-288). The NTD contains a conserved HHCC Zn-coordination motif, and Zn-binding contributes to IN multimerization and catalytic function [11,12]. The CCD contains an invariant triad of acidic residues (Asp-64, Asp-116, Glu-152 of HIV-1) that forms the enzyme active site [13-16]. The CCD also contributes to IN multimerization [17] and engages viral [18-20] and chromosomal [21,22] DNAs during integration. The CTD, which is the least conserved of the domains among retroviruses [23], also contributes to specific [24] and non-specific [25-27] DNA interactions, as well as multimerization [28].

Insight into the mechanism of HIV-1 integration is somewhat hampered by lack of relevant 3-dimensional information, as structures for the enzyme bound to its DNA substrates, or the free holoenzyme, have yet to be reported. NTD-CCD [29-31] and CCD-CTD [32-34] two-domain x-ray crystal structures have nevertheless been informative. Three NTD-CCD structures, containing HIV-1, HIV-2, or maedi-visna virus domains, have revealed a dimer-of-dimers architecture for the active IN tetramer [29,30] and the high affinity binding mode of the common lentiviral integration cofactor LEDGFp75 [31]. An SH3 fold comprised of five β strands makes up the heart of the CTD [35,36], and a comparison of HIV-1 [32], SIV [33], and Rous sarcoma virus [34] CCD-CTD structures reveals considerable flexibility in CTD positioning with respect to the different CCDs. Nevertheless, extended viral DNA binding surfaces were ascribed to each CCD-CTD structure. Although residues 271-288, herein referred to as the tail, were present in the two-domain HIV-1 construct, they were disordered and therefore unseen in the resulting crystal structure [32].

The roles of the C-terminal tail in IN function and HIV-1 replication are largely unexplored. The IN_1-270 _deletion mutant that lacked the tail supported 10-50% of wild-type (WT) Mn^2+^-dependent 3' processing and DNA strand transfer activities, whereas the activities of IN_1-279 _were largely unimpaired (50-100% of WT) [25]. HIV-1 carrying the substitution of Ala for Lys-273 grew like the WT in Jurkat T cells, dispensing an obvious role for this highly conserved tail residue in virus replication [37]. To learn more about the role of this region in IN catalysis and HIV-1 replication, successive three amino acid deletion mutants were constructed and analyzed in various enzymatic and virus infection assays. The somewhat larger 23-residue HIV-2 tail was moreover swapped for the HIV-1 sequence to assess the activities of tail chimera enzyme and virus., C-terminal deletion mutants that lack all or part of the tail could be useful structural biology candidates due to their inability to adopt an ordered fold in previous crystal structures. Thus, one goal of this study was to evaluate the solubility-enhancing F185K CCD mutation [38] for its potential effects on the in vitro activities of tail deletion mutant enzymes.

## Methods

### Plasmid DNA constructions

Bacterial expression vector pKBIN6Hthr [39] and viral IN shuttle vector pUCWTpol [40] were previously described. Because the IN tail overlaps the 5' end of *vif*, shuttle vector pUCWTpol3stop, which harbored three stop codons after Vif residue Asn-19, was constructed by PCR using Pfu Ultra DNA polymerase (Stratagene, La Jolla, CA) and primers AE1064 (5'-ACAGGATGAGGATTAACTGATGATAAGCTTTAGTAAAACACCATATG)/AE1065 (5'-CATATGGTGTTTTACTAAAGCTTATCATCAGTTAATCCTCATCCTGTC). IN deletion mutations were subsequently constructed in pUCWTpol3stop or pKBIN6Hthr by PCR. Plasmid pUCWTpolBam-Spe, which contains unique BamHI and SpeI sites downstream of the IN coding region and a stop codon after Arg-17 in Vif [41], was used to swap tail sequences as follows. AAA/CAG/ATG, which encodes for HIV-1 residues Lys-273, Gln-274, and Met-275, was changed to GGT/CGA/CTG to imbed a unique SalI site in pUCWTpolSal-Bam-Spe at the HIV-1/2 tail boundary. A linker constructed by annealing AE3697 (5'-PO_4_-TCGACAGGAGATGGACAGCGGAAGTCACCTGGAGGGCGCAAGAGAGGACGGTGAGATGGCATAAG) with AE3698 (5'-PO_4_-GATCCTTATGCCATCTCACCGTCCTCTCTTGCGCCCTCCAGGTGACTTCCGCTGTCCATCTCCTG) was then ligated to SalI/BamHI-digested pUCWTpolSal-Bam-Spe. To move the chimera tail to pKBIN6Hthr, pUCWTpolSal-Bam-Spe was amplified using XhoI-tagged AE3699 (5'-TGGTGCTCGAGTGCGGACCCACGCGGGACGAGTGCCATCTCACCGTCCTCTCTTGC) and AflII-tagged AE3700 (AACATCTTAAGACAGCAGTAC) and the resulting digested fragment was ligated with XhoI/AflII-cut pKBIN6Hthr. Mutated AgeI-PflMI 1.8 kb fragments from pUCWTpol3stop or pUCWTpolSal-Bam-Spe were swapped for the corresponding fragment in the single round HIV-1_NL4-3_-based vector pNLX.Luc(R-) [42]. All plasmid regions constructed by PCR were analyzed by DNA sequencing to verify targeted changes and lack of unwanted secondary mutations.

### Protein expression and purification

*Escherichia coli *strain PC2 [43] transformed with IN expression constructs were grown for 16 h at 30°C. The next day bacteria subcultured at 1:30 in 600 ml LB-100 μg/ml ampicillin were grown at 30°C until A_600 _of 0.6, at which time expression was induced by the addition of 0.6 mM isopropyl-β-D-thiogalactopyranoside. Cells were harvested following 5 h of induction at 28°C. The bacterial pellet resuspended in ice-cold buffer A [25 mM Tris-HCl, pH 7.4, 1 M NaCl, 7.5 mM 3-[(3-Cholamidopropyl)dimethylammonio]-2-hydroxy-1-propanesulfonate (CHAPS)] containing 25 mM imidazole-0.5 mM phenylmethanesulphonylfluoride was sonicated. After centrifugation for 30 min at 39,000 *g*, the supernatant was incubated with 0.6 ml of buffer A-25 mM imidazole-equilibrated Ni^2+^-nitrilotriacetic acid (Ni-NTA) agarose beads (QIAGEN, Valencia, CA) at 4°C for 3 h. The beads were washed twice with 20 volumes of buffer A-25 mM imidazole followed by washing with 30 volumes of buffer A-35 mM imidazole. IN-His_6 _was eluted with buffer A-200 mM imidazole. IN containing fractions identified by Na dodecyl sulfate (SDS)-polyacrylamide gel electrophoresis were pooled and dialyzed overnight against buffer D [25 mM Tris-HCl, pH 7.4, 1 M NaCl, 7.5 mM CHAPS, 10% glycerol (w/v), 10 mM dithiothreitol (DTT)]. The His-Tag was removed using 40 U of thrombin (Sigma-Aldrich, St. Louis, MO) per mg of protein for 3 h at room temperature, which left the heterologous LVPR sequence at each C-terminus. After removal of thrombin by incubation with Benzamidine beads (Novagen, Madison, WI), IN was concentrated using Centricon-10 Concentrators (Millipore, Billerica, MA) and dialyzed against buffer D for 4 h. Protein concentration was determined by spectrophotometer, and aliquots flash frozen in liquid N_2 _were stored at -80°C. Quantitative image analysis (Alpha Innotech FlourChem FC2, San Leandro, CA) of Coomassie-stained gels revealed that each IN preparation was minimally 90% pure.

Recombinant LEDGFp75 expressed in bacteria was purified as previously described [44]. LEDGFp75 concentrations were determined using the Bio-Rad protein assay kit (Hercules, CA). Exonuclease III was from New England Biolabs (Beverley, MA).

Anti-IN monoclonal antibody 8G4 [45] was purified from hybridoma cell supernatant using protein G sepharose (GE Healthcare, Piscataway, NJ) following the manufacturer's recommendations. 500 ml of cell supernatant loaded onto 1 ml of protein G beads were subsequently washed with phosphate-buffered saline. Antibody eluted with 20 mM glycine-HCl, pH 2.8 was immediately neutralized by addition of 1 M Tris-HCl, pH 8.5. Pooled fractions were concentrated by ultrafiltration, and resulting antibody concentration was determined by spectrophotometry.

### In vitro integration assays

Oligonucleotides that mimic the HIV-1 U5 end were used as viral DNA substrates. AE143 (5'-ACTGCTAGAGATTTTCCACACTGACTAAAA) and AE191 (5'-TTTTAGTCAGTGTGGAAAATCTCTAGCAG) were annealed prior to filling-in the 3' recess with [α-^32^P]TTP (3000 Ci/mmol; PerkinElmer, Waltham, MA) using Sequenase version 2.0 T7 DNA polymerase (GE Healthcare) to label the phosphodiester within the pGT_OH _dinucleotide that is cleaved during 3' processing [3,46]. To prepare a 30 bp preprocessed duplex for DNA strand transfer, AE155 (5'-TTTTAGTCAGTGTGGAAAATCTCTAGCA) 5'-end labeled with [γ-^32^P]ATP (3000 Ci/mmol; PerkinElmer) using T4 polynucleotide kinase (GE Healthcare) [46] was annealed with AE143. Unincorporated radionuclide was removed by passing labeled duplexes through Bio-Spin 6 columns (Bio-Rad) equilibrated with 10 mM Tris-HCl, pH 8.0-20 mM NaCl-0.1 mM EDTA.

Reaction mixtures (16 μl) contained 25 mM MOPS, pH 7.2, 10 mM DTT, 31 mM NaCl, 10 mM MgCl_2_, 5 μM ZnSO_4_, 5 nM DNA substrate, and 0.49 μM IN. Reactions stopped by addition of an equal volume of sequencing gel sample buffer (95% formamide, 10 mM EDTA, 0.003% xylene cyanol, 0.003% bromophenol blue) were boiled for 2 min prior to fractionation through 20% polyacrylamide- (3' processing) or 15% polyacrylamide-8.3 M urea (DNA strand transfer) sequencing gels. Reaction products in wet gels exposed to phosphor image plates were quantified using Image Quant version 1.2 (GE Healthcare).

LEDGFp75-dependent concerted integration activity was assayed essentially as previously described [31]. A preprocessed 32 bp U5 end was prepared by annealing AE3653 (5'-CCTTTTAGTCAGTGTGGAAAATCTCTAGCA) with AE3652 (5'- ACTGCTAGAGATTTTCCACACTGACTAAAAGG). Reactions (36 μl) were initiated by mixing 0.5 μM HIV-1 DNA with 0.33 μg pGEM-3 target DNA in 25.3 mM NaCl, 5.5 mM MgSO_4_, 11 mM DTT, 4.4 μM ZnCl_2_, 22 mM HEPES-NaOH, pH 7.4. IN (2 μl) in dilution buffer (750 mM NaCl, 10 mM DTT, 25 mM Tris-HCl, pH 7.4) was then added. Following 2-3 min at room temperature, 2.0 μl of LEDGFp75 was added, and the reactions were allowed to proceed at 37°C for 1 h. The final concentrations of IN and LEDGFp75 were both 0.8 μM. Reactions stopped by the addition of EDTA and SDS to the final concentrations of 25 mM and 0.5%, respectively, were deproteinized using 30 μg proteinase K (Roche Molecular Biochemicals, Indianapolis, IN) for 60 min at 37°C. DNAs recovered following precipitation with ethanol were separated on 1.5% agarose-TAE (40 mM Tris base, 20 mM acetate, 1 mM EDTA) gels run in TAE at 150 V for 2 h. DNAs stained with ethidium bromide (0.5 μg/ml) were quantified using Alpha Innotech FlourChem FC2.

### Cells and viruses

293T cells were grown in Dulbecco's modified Eagle's medium (DMEM) supplemented to contain 10% fetal bovine serum (FBS) (Invitrogen Corporation, Carlsbad, CA). Cells were plated at 8.6 × 10^6^/10-cm dish 24 h prior to transfection. Virus stocks were prepared by co-transfecting cells with 10 μg pNLX.Luc(R-) and 1 μg of envelope expression vector pCG-VSV-G [47] using FuGene 6 as described by the manufacturer (Roche Molecular Biochemicals). Cell-free supernatants harvested at 48 h post-transfection were passed through 0.45 μm filters. Virus titer was determined using an exogenous reverse transcriptase (RT) assay as previously described [48]. For western blot analysis, viruses pelletted by ultracentrifugation at 122,000 *g *for 2 h at 4°C were lysed for 15 min on ice in 40 μl of buffer containing 140 mM NaCl, 8 mM Na_2_HPO_4_, 2 mM NaH_2_PO_4_, 1% Nonidet P40, 0.5% Na deoxycholate, 0.05% SDS. Supernatant recovered after centrifugation at 19,800 *g *was stored at -80°C. Following electrophoresis and transfer to polyvinylidene fluoride, IN and p24 were detected using 1:100 and 1:5000 dilutions of 8G4 and 13-203-000 (Advanced Biotechnologies Inc, Columbia, MD) antibodies, respectively.

HeLa-T4 cells [49] were grown in DMEM-10% FBS containing 100 IU/ml penicillin and 100 μg/ml streptomycin. For infectivity measurements, cells plated at 75,000 cells/well of 24-well tissue culture plates 24 h prior to infection were incubated in duplicate with 10^6 ^RT-cpm of virus for 17 h, after which cells washed with phosphate-buffered saline were replenished with fresh media. At 46 h post-infection, cells were collected, washed, and lysed using 75 μl passive lysis buffer as recommended by the manufacturer (Promega Corp., Madison, WI). Luciferase activities (20 μl), determined in duplicate for each infection, were normalized to total levels of cellular protein as previously described [42]. For quantitative (Q)-PCR assays, 900,000 cells were plated per 10 cm dish the day before infection. Cells were infected with 2.3 × 10^7 ^RT-cpm of TURBO DNase-treated [42] native or heat-inactivated (65°C for 30 min) virus. 8G4 hybridoma cells were grown in DMEM containing 10% ultra low IgG FBS (Invitrogen Corporation) with penicillin and streptomycin.

### Q-PCR assays for reverse transcription and integration

Total cellular DNA was isolated at 7 or 24 h post-infection using the QIAamp DNA mini kit (QIAGEN). Late reverse transcription (LRT) products were detected using primers and Taqman probe as previously described [50,51]. Two-long terminal repeat (2-LTR) containing circles were detected at 24 h post-infection using primers MH535/536 [50] and SYBR green (QIAGEN). Integration was measured at 24 h using a modified nested HIV-1 R-Alu format based on reference [52]. DNA (100 ng) was amplified using the phage lambda T-R chimera primer AE3014 [53] and Alu-specific AE1066 (5'-TCCCAGCTACTCGGGAGGCTGAGG) with rTth DNA polymerase XL as recommended by the manufacturer (Applied Biosystems Inc, Foster City, CA). Samples (1 μl) were then analyzed by Q-PCR using SYBR green with primers AE989 and AE990 [51]. DNA generated from WT-infected cells was end-point diluted in DNA prepared from uninfected cells to generate the integration standard curve. LRT, 2-LTR, and Alu-integration Q-PCR values obtained from samples prepared using heat-inactivated virus were subtracted from those generated using native virus.

## Results and Discussion

### Experimental strategy

**Figure 1:**
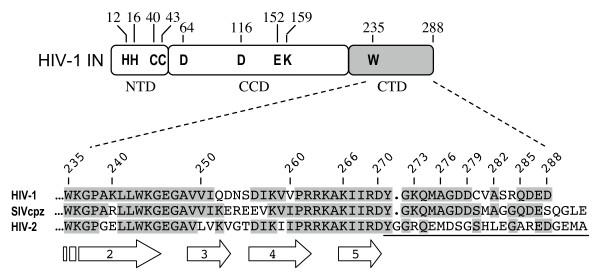
**IN sequence alignment and HIV-1 mutants analyzed in this study**. The upper drawing indicates the three IN domains, with amino acid residues conserved among all retroviruses noted. CTD sequences downstream of the invariant Trp are shown below for HIV-1 (NL4-3 isolate, accession number M19921), SIVcpz (accession number AF115393), and HIV-2 (ROD isolate, accession number M15390). Residues that appear in more than one sequence are highlighted in grey. The broad arrows beneath the alignment indicate the β strands that comprise the SH3 fold [35,36]. Numbers 266-285 above the alignment mark the IN deletion mutant enzymes and viruses analyzed in this study. The underline indicates the region of HIV-2 IN that was swapped for HIV-1 residues 271-288.

Little is known about the role of HIV-1 IN C-terminal tail (residues 271-288, Figure 1) in integration. This region of the protein, which overlaps the 5' end of the *vif *reading frame, is fairly well conserved among different HIV-1 isolates. Some clade C sequences harbor Ala in place of Asp-278 and numerous clades as well as SIVcpz carry Gly at position 283 (Figure 1); the remaining residues by contrast show little or no sequence variation [54]. To ascertain the role of the tail in IN function, six nested deletions mutants lacking 3, 6, 9, 12, 15, or 18 amino acids from the C-terminus were constructed in the pKBIN6Hthr bacterial expression construct [39] and luciferase-based pNLX.Luc(R-) viral vector [42] (Figure 1). The CCD F185K mutation, which dramatically increases the solubility of the HIV-1 protein [38], was tested in some constructs to assess its potential affects on IN activities in vitro. The 1-266 deletion mutant, which lacked the C-terminal 22 residues and hence the fifth β strand of the CTD SH3 fold in addition to the tail (Figure 1) [35,36], was used as a loss-of-function control [55]. Finally, the 23 residue HIV-2 tail (underlined in Figure 1) was swapped for the corresponding HIV-1 sequence to test the functionality of this marginally related sequence substitution. Because the viral changes necessarily altered the overlapping *vif *sequence, these constructs incorporated stop codons downstream of the IN region within the *vif *frame to negate synthesis of altered Vif proteins. Viruses were constructed in 293T cells, which lack APOBEC3G and thus do not require functional Vif to yield infectious particles [56].

### The C-terminal tail and IN enzymatic activities

**Figure 2:**
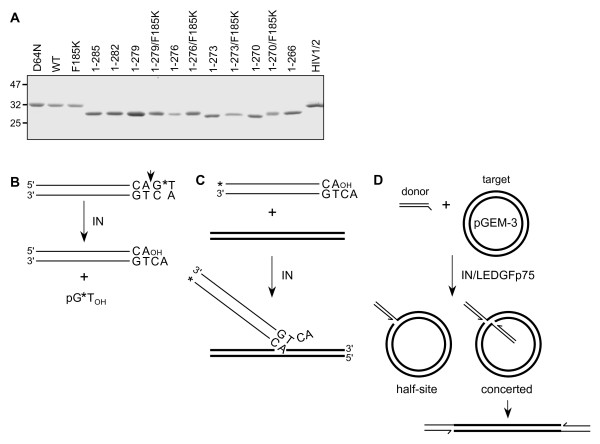
**Integrase proteins and in vitro integration assays**. (A) Purified proteins (approximately 5 μg each) were stained with Coomassie blue following SDS-polyacrylamide gel electrophoresis. Migration positions of molecular mass standards in kDa are shown on the left. (B) 3' Processing assay. The blunt-ended viral DNA substrate is shown highlighting the subterminal CA that is conserved among all retroviruses, retrotransposons, and some bacterial transposases. During 3' processing, IN cleaves the A/G phosphodiester bond (short vertical arrow), releasing radiolabelled pGT_OH _dinucleotide. (C) The DNA strand transfer assay utilizes a preprocessed viral DNA end. Integration into target DNA yields products whose lengths exceed that of the starting substrate. (D) Two different DNAs, viral donor (oligonucleotide drawn in the same orientation as in panel C, top) and circular target, are used in the concerted integration assay. In the presence of LEDGFp75, some donor DNA is integrated into only one strand of the target to yield a tagged, nicked circle half-site reaction product. Concerted integration across the major groove by contrast yields a linearized product whose length exceeds that of the starting circle by twice the length of the viral donor. For panels B-D, thin and bold lines represent viral donor and target DNAs, respectively. *, positions of ^32^P label (panels B and C).

Recombinant proteins were engineered to contain C-terminal hexahistidine tags to facilitate purification. Though this might appear counterintuitive given the C-terminal focus of the study, it was necessary to obtain relatively pure preparations. The tail region is hypersensitive to proteolysis during expression in *E. coli *[57], and preliminary experiments with N-terminally tagged proteins yielded heterogeneous populations eluted from Ni-NTA beads whose purities were not substantially improved upon by subsequent ion exchange or size exclusion chromatography (data not shown). The C-terminal tag obviated this problem, as proteolyzed variants failed to bind Ni-NTA beads. Indeed, quantitative image analysis of purified WT and mutant proteins revealed near homogeneous preparations (Figure 2A).

IN activities were measured using three different assay designs, each of which incorporated an ~30 bp DNA mimic of the viral U5 end (Figure 2B-D). Overall levels of IN 3' processing and DNA strand transfer activities were determined in two separate assays using differentially labeled 30 bp substrates (Figure 2B and 2C). Under these conditions, the majority of DNA strand transfer reaction products result from the insertion of a single oligonucleotide end into one strand of a second target DNA molecule [8]. By contrast, integration in cells proceeds via the concerted insertion of viral U3 and U5 DNA ends into opposing strands of chromosomal DNA. Reactions that contain relatively low concentrations of IN protein [58], relatively long viral DNA substrates [59], or relatively high concentrations of oligonucleotide substrate in the presence of LEDGFp75 [31] support efficient concerted HIV-1 integration. Here, LEDGFp75 was used in a third assay format (Figure 2D) to monitor the concerted integration activities of IN mutant proteins. His_6_-tags were removed from purified IN proteins by thrombin cleavage prior to enzyme assays, yielding the remnant LVPR C-terminal sequence. Experiments conducted with a subset of proteins prior to cleavage (WT, 1-279, 1-273, 1-270,1-266, and HIV-1/2) revealed similar levels of 3' processing activities relative to WT, indicating that the remnant sequence did not significantly influence mutant enzyme activities (data not shown).

**Figure 3:**
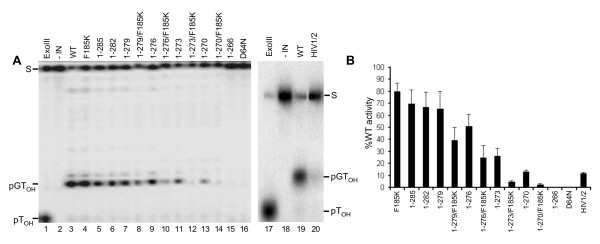
**WT and mutant IN 3' processing activities**. (A) Polyacrylamide gel images reveal the migration positions of labeled 30-mer DNA substrate (S), cleaved pGT_OH _dinucleotide, as well as pT_OH _mononucleotide. The reactions loaded in lanes 1 and 17 contained exonuclease III in place of IN, whereas lanes 2 and 18 omitted IN. The reactions in the remaining lanes contained the indicated IN proteins. (B) Mutant 3' processing activities plotted as percentage of WT IN function. Results are mean ± SEM for two (HIV-1/2 chimera) to four (all other mutants) independent experiments.

To follow the course of the 3' processing reaction, oligonucleotide substrate DNA was labeled at the inter-nucleotide linkage of the 3'-terminal GT (Figure 2B); IN mediated hydrolysis liberates pGT_OH_, which is readily distinguished from the 30 bp substrate following electrophoresis on high percentage DNA sequencing gels [3,4] (Figure 3A, lanes 2 and 3; results quantified in panel B). Exonuclease III-mediated hydrolysis by contrast yielded free pT_OH _(Figure 3A, lanes 1 and 17). All IN preparations were basically void of contaminating exonuclease activity (Figure 3A), reflecting the relatively high degrees of protein purity (Figure 2A). IN_D64N _and IN_1-266_, which contained the substitution of Asn for active site residue Asp-64 [14] and lacked part of the CTD SH3 fold, respectively, were predictably inactive (Figure 3A, lanes 15 and 16). The activities of the three mutants that retained most of the tail, IN_1-285_, IN_1-282_, and IN_1-279_, were overall similar at 65-70% of WT (Figure 3A, lanes 5-7). Mutants with further progressive tail deletions yielded a stepwise reduction in 3' processing activity, as IN_1-276_, IN_1-273_, and IN_1-270 _supported about 51%, 26%, and 13%, respectively, of WT function. Thus, IN_1-279 _and IN_1-270 _support Mg^2+^-dependent 3' processing activities that do not significantly differ from those reported using Mn^2+ ^[25]. The IN_HIV1/2 _chimera protein like IN_1-270 _retained marginal (about 12% of WT) activity (Figure 3A, lane 20; Figure 3B). The F185K solubility mutation marginally impacted activity, generally yielding 20-25% reductions when compared to the same protein lacking the CCD change (Figure 3B).

**Figure 4:**
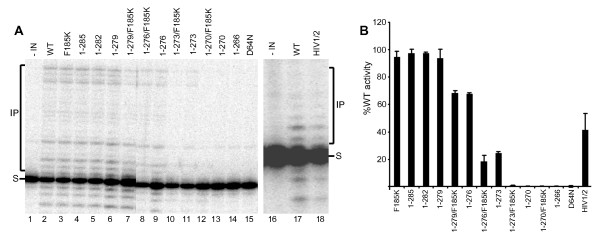
**IN mutant DNA strand transfer activities**. (A) Scanned gel images show the migration positions of preprocessed substrate (S) DNA as well as the integration products (IP) of DNA strand transfer. IN was omitted from the reactions loaded in lanes 1 and 16; the remaining lanes contained the indicated IN proteins. (B) Mean DNA strand transfer activities ± SEM for two independent experiments plotted as percentage of WT IN activity.

The preprocessed DNA strand transfer substrate was labeled at the 5' end of the strand that becomes joined to target DNA; IN activity yields a population of products that migrate more slowly than the starting substrate on DNA sequencing gels [8] (Figure 2C and 4A). Relative levels of IN mutant DNA strand transfer activities in large part mirrored 3' processing activities with some subtle differences noted (compare Figure 4B to Figure 3B). IN_1-285_, IN_1-279_, and IN_1-276 _supported DNA strand transfer at basically the same level as the WT, whereas the activity of IN_1-270 _was undetectable (Figure 4A, lanes 4-6 and 13; Figure 4B). Mn^2+^can support more robust IN activity than Mg^2+ ^[9,60], which may have contributed to the previously reported residual level of IN_1-270 _DNA strand transfer activity [25]. IN_HIV1/2 _DNA strand transfer activity, by contrast to IN_1-270_, was increased from its relative level of 3' processing activity (Figure 4B and 3B).

**Figure 5:**
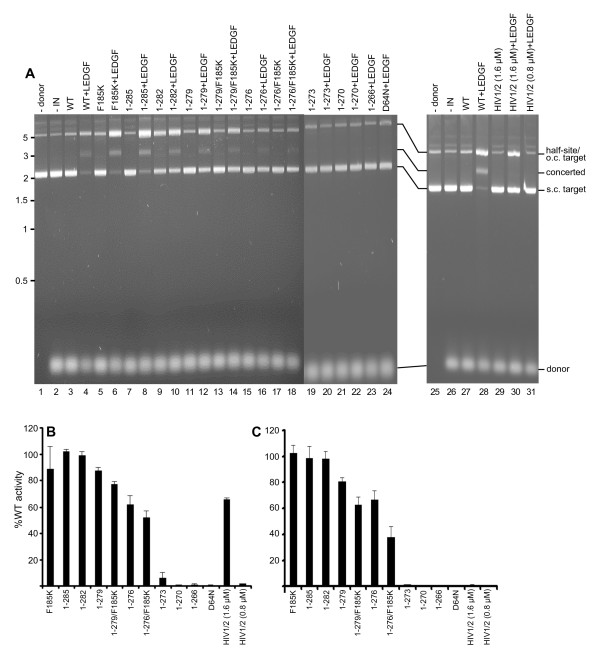
**LEDGFp75-dependent concerted integration activities of WT and IN mutant proteins**. (A) The scanned ethidium-stained agarose gels reveal the migration positions of donor, supercoiled (s.c.), and open circular (o.c.) substrate DNAs, as well as half-site and concerted integration reaction products. Donor DNA was omitted from the reactions analyzed in lanes 1 and 25, whereas IN was omitted from lanes 2 and 26. The remaining lanes contained the indicated IN proteins and, at times, LEDGFp75. The concentration of HIV-1/2 IN in lanes 29 and 30 was 1.6 μM, whereas all other IN concentrations were 0.8 μM. The migration positions of molecular mass standards in kb are shown to the left of the gel. (B and C) Levels of overall and concerted DNA strand transfer activities, respectively, normalized to IN_WT _(set to 100%). Results are mean ± SEM for two independent experiments.

Supercoiled pGEM-3 plasmid DNA was incorporated into the reaction mixture to help identify concerted integration reaction products (Figure 2D and 5A). Integration of only one donor DNA end into one plasmid DNA strand yields a tagged circle whose mobility through agarose matches that of starting relaxed circular plasmid (Figure 5A). Pairwise integration of two oligonucleotides by contrast yields a linearized product whose size is slightly larger than linear plasmid (Figure 2D). IN DNA strand transfer activity was barely detectable in the absence of LEDGFp75, yielding slight increases in the nicked or open circular plasmid population (Figure 5A, compare lanes 3 and 27 to lanes 2 and 26, respectively) [31]. LEDGFp75 greatly stimulated IN activity such that the supercoiled target DNA was largely consumed, yielding a mixture of half-site and concerted integration products (Figure 5A, lanes 4 and 28). IN mutant product formation was quantified to reflect overall levels (half-site plus concerted, Figure 5B) of DNA strand transfer activities or just concerted integration (Figure 5C). The overall activities of the various deletion mutant proteins in large part mirrored their oligonucleotide-based DNA strand transfer activities (compare Figure 5B to 4B). Though 0.49 μM IN_HIV1/2 _supported about 40% of IN_WT _activity in the oligonucleotide-based assay (Figure 4B), 0.8 μM protein failed to support appreciable product formation in the concerted assay format (Figure 5A, lane 31). Doubling the amount of input IN_HIV1/2 _to 1.6 μM yielded significant half-site product formation (about 66% of IN_WT_, Figure 5A, lane 30 and Figure 5B) in the absence of detectable concerted integration activity (Figure 5C). Taken together, our data indicate that the C-terminal tail does not play a specific role in concerted DNA integration, though the introduction of a foreign sequence for the HIV-1 tail can uncouple pairwise from single end integration activity. Though others noted that the F185K substitution ablated Mg^2+^-dependent integration of preprocessed oligonucleotide donor DNA into heterologous target DNA [61], our reaction conditions failed to reveal an affect of the solubilizing mutation on full-length IN activity in the presence of LEDGFp75 (Figure 5A, lane 6; panels B and C). We furthermore conclude that the C-terminal 9 amino acids of HIV-1 IN can be removed without dramatically effecting Mg^2+^-based single end or concerted DNA integration activities (Figures 3, 4, 5)., We highlight these derivatives as potential candidates for structural biology studies despite the approximate 20-25% reductions in IN_1-279 _and IN_1-276 _activities brought on by the F185K change. We would by contrast advise against extensive analysis of tailless IN_1-270_, due to its lack of detectable DNA strand transfer activity under these assay conditions (Figure 4 and 5).

### Characterization of IN mutant viruses

**Figure 6:**
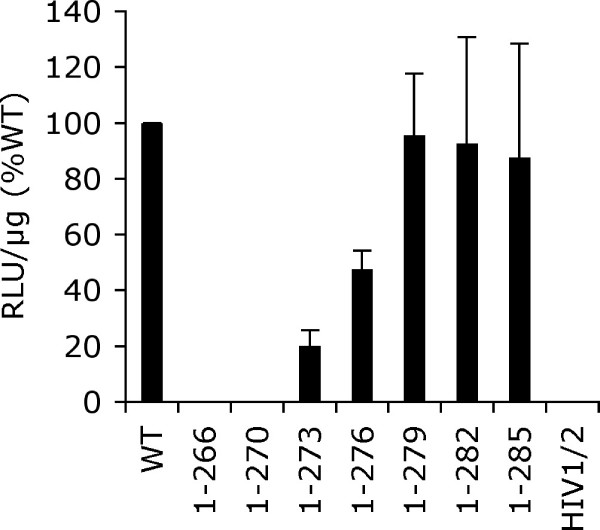
**IN mutant viral infectivity**. Normalized levels of IN mutant infectivities are shown relative to WT HIV-1 (set at 100%). Each experiment amassed duplicate luciferase assays of duplicate infections. Shown is the mean ± SEM of five independent experiments. RLU, relative light units.

To assess HIV-1 infectivity, HeLa-T4 cells were infected with normalized levels of single-round viruses that carry the luciferase reporter gene in place of *nef*. Two days post-infection, cells were harvested and resulting luciferase activities were normalized to the levels of total protein in the different cell extracts [42,47]. Deletion of up to 9 amino acids from the IN C-terminus failed to affect HIV-1 infectivity (Figure 6). IN mutants HIV-1_1-276 _and HIV-1_1-273 _supported about 50% and 20% of the level of WT infection, respectively, whereas HIV-1_1-270_, HIV-1_1-266_, and the HIV-1/2 tail chimera were non-infectious (Figure 6).

**Figure 7:**
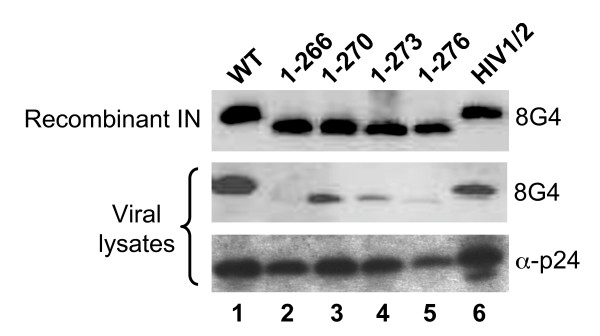
**WT and IN mutant virus protein content**. Top panel, 2 ng of the indicated recombinant IN protein was analyzed by western blotting. Lower panels, viral lysates. The primary blotting antibody is indicated to the right of each panel.

IN mutations can affect multiple steps in the HIV-1 replication cycle, including particle release from virus-producing cells and/or reverse transcription during the subsequent round of infection (reviewed in ref. [62]). Viruses specifically blocked at integration are distinguished as class I, whereas class II mutants display additional stage defects. To assess potential affects on virus particle release, RT content in HeLa cell supernatants at 2 days post-transfection was normalized to levels of cell-associated luciferase activity. Normalized levels of mutant virus release did not significantly differ from the WT under this assay condition (data not shown). Defective mutant viruses (HIV-1_1-266_, HIV-1_1-270_, HIV-1_1-273_, HIV-1_1-276_, and HIV-1/2; Figure 6) produced from transfected 293T cells were analyzed by western blotting to assess levels of virion-incorporated IN protein. Monoclonal antibody 8G4, which recognizes discontinuous epitopes in the NTD and CCD [45], was utilized to avoid potential complications from the CTD mutations. Accordingly, 8G4 effectively recognized the different forms of recombinant IN protein (Figure 7, top panel). Based on relative levels of p24 content (bottom panel), we conclude that HIV-1_1-276_, HIV-1_1-273_, HIV-1_1-270_, and HIV-1_1-266 _harbor significantly less IN protein than WT HIV-1 (viral lysate panels, compare lanes 2-5 to lane 1), with HIV-1_1-266 _suffering the most dramatic defect (lane 2). We therefore conclude that an intact SH3 fold plays an important role in Gag-Pol incorporation and/or IN retention in virions.

**Figure 8:**
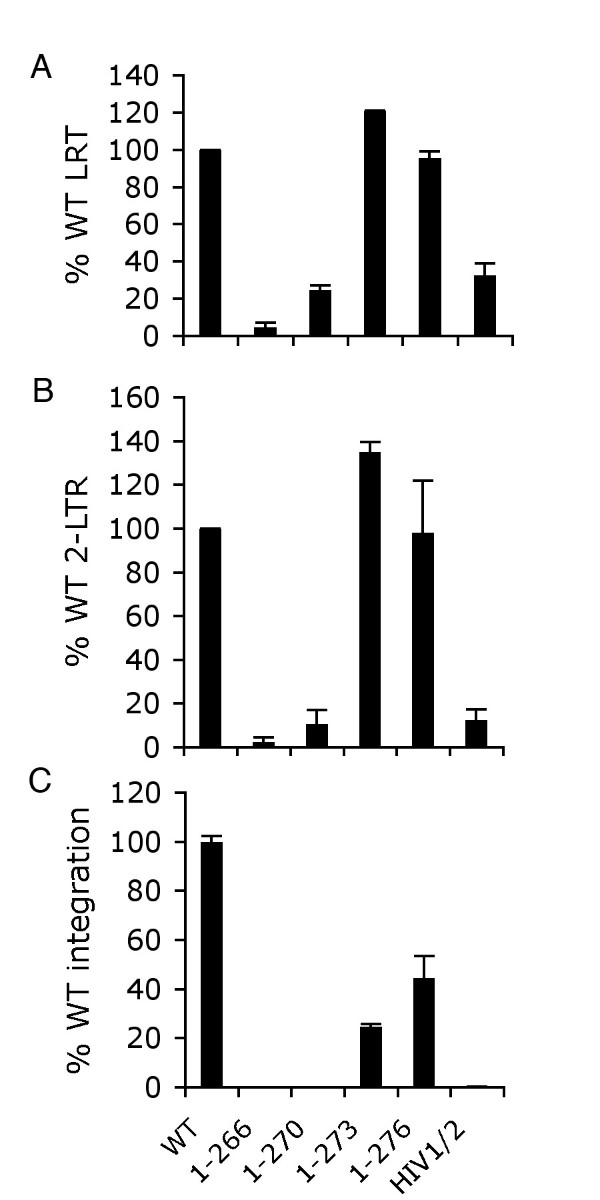
**Reverse transcription and integration profiles of IN mutant viruses**. (A) Mutant viral LRT levels, graphed as percentages of the WT (leftward bar). (B) 2-LTR circle levels at 24 h post-infection. (C) Mutant viral integration in comparison to the WT. Panels A and B average results of two different infection experiments (mean ± SEM). Mean ± SEM of duplicate Q-PCR assays of one infection experiment is shown in panel C. The panel C data are representative of those obtained from a duplicate set of infections.

Q-PCR assays were utilized to assess defective mutant virus reverse transcription (LRT at 7 h post-infection) and 2-LTR circle formation and integration (nested Alu-R PCR) at 24 h. Virus stocks were treated with DNase prior to infection to digest plasmid DNA that may persist after transfection and hence template in the LRT reaction format. To control for potential plasmid carry-over, a parallel set of infections was conducted using heat-inactivated viruses. Resulting LRT values (typically 1-5%) were subtracted from native viral infections. HIV-1_1-276 _and HIV-1_1-273 _supported the WT levels of reverse transcription and circle formation (Figure 8A and 8B), whereas HIV-1_1-270_, HIV-1_1-266_, and the HIV-1/2 chimera supported about 25%, 5%, and 33% of WT LRT product formation (Figure 8A). Under these experimental conditions IN residues 271-273 contribute to reverse transcription. Due to the pleiotropic nature of HIV-1 IN mutations these results were not entirely unexpected. Residues 271-273 might influence the interaction between IN and RT [63], which occurs via the CTD [64,65]. An RT binding interface was recently mapped to β strands 2-4 of the SH3 fold [66] and though residues 271-273 abut β-5 (Figure 1), it is not unreasonable to suspect the disordered tail could affect RT binding. Alternatively, a number of NTD and CCD mutations in addition to CTD changes can impair DNA synthesis (see [62] for review), indicating that the C-terminal tail changes might perturb reverse transcription via global affects on IN and/or the preintegration complex.

HIV-1_1-276 _and HIV-1_1-273 _supported about 40% and 20% of WT integration, respectively (Figure 8C), indicating that their partial infectivities (Figure 6) were due to specific integration defects attributable to the intrinsic activities of the deletion mutant enzymes (Figure 3, 4, 5). Consistent with their non-infectious phenotypes and inabilities for recombinant IN proteins to catalyze concerted integration activity, neither HIV-1_1-270 _nor the HIV-1/2 chimera supported a detectable level of integration during infection (Figure 8C). As both of these viruses supported the formation of detectable 2-LTR circles (Figure 8B), we group them as class II defective IN mutants that display marginal (3 to 4-fold) reverse transcription in additional to prominent integration defects. HIV-1_1-266 _was a more severe class II mutant virus, harboring a significant reverse transcription as well as integration defect.

## Conclusion

The results of this study revealed that nine amino acids can be removed from the HIV-1 IN C-terminus without significantly affecting the activity of the enzyme or infectivity of the virus. Additional removal of up to six amino acids impacted infectivity by up to 80%, yielding viruses that were specifically defective for integration due to the compromised activities of the associated IN_1-276 _and IN_1-273 _enzymes. Heuer and Brown [67] reported that residues 271-288 crosslink to viral and target DNA sequences within junctional disintegration substrates. We would therefore surmise that tail residues 271-279 interact with substrate DNA during integration. HIV-1_1-270 _was non-infectious and harbored an approximate fourfold reverse transcription defect. This suggests IN residues 271, 272, and 273 might impact its physical association with RT. HIV-1_1-266_, which lacked the fifth β strand of the fold, failed to incorporate significant levels of IN protein and was in large part defective for reverse transcription. Thus, an intact SH3 fold apparently contributes to Gag-Pol packaging and subsequent viral DNA synthesis. Our results moreover highlight partial tailed variants 1-279/F185K and 1-276/F185K as viable candidates for structural biology studies, as they retained >20% of IN enzymatic activities yet lacked at least half of the disordered region.

## List of abbreviations used

CCD: catalytic core domain; CHAPS: 3-[(3-Cholamidopropyl)dimethylammonio]-2-hydroxy-1-propanesulfonate; CTD: C-terminal domain; DMEM: Dulbecco's modified Eagle's medium; DTT: dithiothreitol; FBS: fetal bovine serum; IN: integrase; LRT: late reverse transcription; LTR: long-terminal repeat; Ni-NTA: Ni^2+^-nitrilotriacetic acid; NTD: N-terminal domain; Q: quantitative; RT: reverse transcriptase; SDS: Na dodecyl sulfate; WT: wild type.

## Competing interests

The authors declare that they have no competing interests.

## Authors' contributions

MJD constructed molecular clones, purified recombinant IN proteins, and conducted in vitro integration assays. BM performed the brunt of virological measurements including infectivity, LRT, and 2-LTR circle Q-PCRs. LK purified 8G4 antibody, performed western blotting, and performed some IN purifications and enzyme assays. MCS performed Alu-PCR and quantified virus release from transfected HeLa cells. FDN devised the western blotting procedure, and trained and supervised BM. DEH supplied essential reagents. AE conceived of the study, supervised and interpreted experimental results, and wrote the manuscript. All authors read and approved the final manuscript.
